# Optimized protocol for the extraction of RNA and DNA from frozen whole blood sample stored in a single EDTA tube

**DOI:** 10.1038/s41598-021-96567-2

**Published:** 2021-08-23

**Authors:** Hirotaka Yamagata, Ayumi Kobayashi, Ryouichi Tsunedomi, Tomoe Seki, Masaaki Kobayashi, Kosuke Hagiwara, Chong Chen, Shusaku Uchida, Go Okada, Manabu Fuchikami, Toshiharu Kamishikiryo, Jun-ichi Iga, Shusuke Numata, Makoto Kinoshita, Takahiro A. Kato, Ryota Hashimoto, Hiroaki Nagano, Yasumasa Okamoto, Shuichi Ueno, Tetsuro Ohmori, Shin Nakagawa

**Affiliations:** 1 Division of Neuropsychiatry, Department of Neuroscience, Yamaguchi University Graduate School of Medicine, 1-1-1 Minami-kogushi, Ube, Yamaguchi 755-8505 Japan; 2 Core Research for Evolutional Science and Technology (CREST), Japan Science and Technology Agency (JST), 4-1-8 Honcho, Kawaguchi, Saitama 332-0012 Japan; 3 Department of Gastroenterological, Breast and Endocrine Surgery, Yamaguchi University Graduate School of Medicine, 1-1-1 Minami-kogushi, Ube, Yamaguchi 755-8505 Japan; 4 SK Project, Medical Innovation Center, Kyoto University Graduate School of Medicine, 53 Shogoin‑Kawahara‑cho, Sakyo‑ku, Kyoto, 606‑8507 Japan; 5 Department of Psychiatry and Neurosciences, Graduate School of Biomedical Sciences, Hiroshima University, 1-2-3 Kasumi, Minami-ku, Hiroshima, 734-8551 Japan; 6 Department of Neuropsychiatry, Molecules and Function, Ehime University Graduate School of Medicine, Shitsukawa, Toon, Ehime 791-0295 Japan; 7 Department of Psychiatry, Graduate School of Biomedical Sciences, Tokushima University, 3-18-5 Kuramoto-cho, Tokushima, 770-8503 Japan; 8 Department of Neuropsychiatry, Graduate School of Medical Sciences, Kyushu University, 3-1-1 Maidashi Higashi-Ku, Fukuoka, 812-8582 Japan; 9 Department of Pathology of Mental Diseases, National Institute of Mental Health, National Center of Neurology and Psychiatry, 4-1-1 Ogawa-Higashi, Kodaira, Tokyo 187-8553 Japan

**Keywords:** Gene expression analysis, Isolation, separation and purification, Gene expression, Gene regulation in immune cells, RNA quality control

## Abstract

Cryopreservation of whole blood is useful for DNA collection, and clinical and basic research. Blood samples in ethylenediaminetetraacetic acid disodium salt (EDTA) tubes stored at − 80 °C are suitable for DNA extraction, but not for high-quality RNA extraction. Herein, a new methodology for high-quality RNA extraction from human blood samples is described. Quickly thawing frozen whole blood on aluminum blocks at room temperature could minimize RNA degradation, and improve RNA yield and quality compared with thawing the samples in a 37 °C water bath. Furthermore, the use of the NucleoSpin RNA kit increased RNA yield by fivefold compared with the PAXgene Blood RNA Kit. Thawing blood samples on aluminum blocks significantly increased the DNA yield by ~ 20% compared with thawing in a 37 °C water bath or on ice. Moreover, by thawing on aluminum blocks and using the NucleoSpin RNA and QIAamp DNA Blood kits, the extraction of RNA and DNA of sufficient quality and quantity was achieved from frozen EDTA whole blood samples that were stored for up to 8.5 years. Thus, extracting RNA from frozen whole blood in EDTA tubes after long-term storage is feasible. These findings may help advance gene expression analysis, as well as biomarker research for various diseases.

## Introduction

Cryopreserved blood samples are mainly used for DNA extraction and analysis^1^, such as gene mutation detection, and are particularly important for biobanks. Blood samples are frequently collected and cryopreserved for clinical and basic research in many facilities, including our laboratory^2^, because the collection procedure is simple and relatively noninvasive. For example, research on blood biomarkers has been vigorously conducted for various diseases, including psychiatric disorders and cancers. Particularly, in psychiatric disorders, blood analysis is an important assessment tool as the central nervous system is difficult to evaluate^3^. Furthermore, it has been suggested that gene expression and DNA methylation patterns in the peripheral blood and brain are to some extent correlated^4^. In addition, it may be necessary to analyze samples from several different facilities to ensure a large sample size, as RNA and DNA isolation and analysis are more clinically efficient and cost effective when performed on a large number of samples. The extraction of RNA and DNA from the same blood sample enables to study the relationship between the methylation of promoter regions of DNA and mRNA expression. Therefore, it is desirable to establish a highly reproducible method for the coextraction of pure RNA and DNA from blood samples.

Although several products for isolating RNA are available, most purification kits are designed for fresh blood samples. If blood is to be cryopreserved for RNA purification, it is recommended that peripheral leukocytes be removed before storage to eliminate RNase activity^5–8^ and/or that blood be stored with RNA-stabilizing reagents, such as the PAXgene blood RNA tubes to prevent RNA degradation^8–11^. Without these precautions, it is difficult to extract intact RNA from cryopreserved whole blood samples in ethylenediaminetetraacetic acid disodium salt (EDTA) collection tubes. This is because freeze/thaw cycles invariably damage the plasma membrane^12,13^, and RNA is degraded by RNases that leak from the ruptured cells and into the plasma. However, RNA-stabilizing reagents are expensive and not always available in general clinics. Moreover, removing leukocytes is a cumbersome procedure that requires specialized equipment, such as a centrifuge and clean bench, which are generally not available in clinics. When thawing frozen samples, the most common method is to thaw them quickly in a water bath at 37 °C^7,8,14–16^. Several research groups have attempted to extract RNA from blood cryopreserved in EDTA tubes^17,18^. However, there are only a few studies comparing the differences in RNA degradation depending on the thawing conditions^15^, and methods for extracting RNA from frozen blood without pretreatment have not been well established.

Freezing induces DNA strand breaks in cells^19^; thus, DNA extraction yield has also been reported to be reduced upon freezing and thawing^20^. When frozen blood samples stored for DNA extraction are donated from different institutions, the amount of DNA extracted might decrease when the blood has already been used for RNA extraction. Increasing the amount of DNA to be extracted without compromising its quality would make it easier for institutions to provide samples.

**Figure 1:**
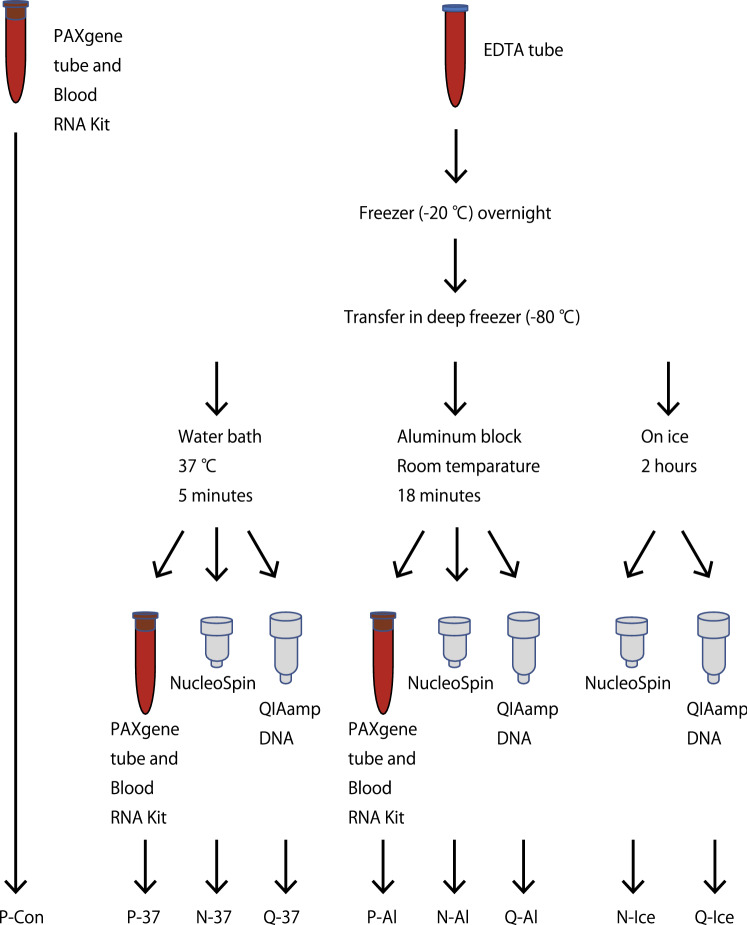
Workflow of RNA and DNA purification in this study. RNA quality and yield under the following six conditions: P-Con, fresh blood and PAXgene kit; P-37, frozen blood thawed using a 37 °C water bath and PAXgene kit; P-Al, thawed using aluminum blocks and PAXgene kit; N-37, thawed using a 37 °C water bath and NucleoSpin kit; N-Al, thawed using aluminum blocks and NucleoSpin kit; and N-Ice, thawed on ice and NucleoSpin kit. DNA yield under the following three thawing conditions using the QIAamp DNA blood kit: Q-37, thawed using a 37 °C water bath; Q-Al, thawed using aluminum blocks; Q-Ice, thawed on ice. The images were created using Microsoft PowerPoint 2016 MSO (16.0.14131.20278) 32 bit (https://www.microsoft.com/) and Adobe Illustrator CS5 (https://www.adobe.com/).

The primary aim of this study was to establish a methodology for extracting high-quality RNA from human blood samples. We compared RNA quality and yield under the following six conditions: P-Con, fresh blood and the PAXgene kit; P-37, frozen blood thawed using a 37 °C water bath and the PAXgene kit; P-Al, thawed using aluminum blocks and the PAXgene kit; N-37, thawed using a 37 °C water bath and the NucleoSpin kit; N-Al, thawed using aluminum blocks and the NucleoSpin kit; and N-Ice, thawed on ice and the NucleoSpin kit (Fig. 1).

The secondary focus was to increase the yield of high-quality DNA extraction from frozen blood. We compared DNA yield under the following three thawing conditions using the QIAamp DNA blood kit: Q-37, thawed using a 37 °C water bath; Q-Al, thawed using aluminum blocks; and Q-Ice, thawed on ice (Fig. 1).

The thawing method using an aluminum block for the isolation of high-quality DNA and RNA from frozen blood samples may help in the molecular characterization of genetic and transcriptional processes and may be applied to more efficiently explore biomarkers in human diseases.

## Results

### RNA quantity and quality

RNA was purified from blood samples (Participants1, *n* = 7) using six different protocols after short-term (48–72 h) storage (Fig. 1). The RNA yields, ratios of the absorbance at 260 and 280 nm (A_260/280_) and at 260 and 230 nm (A_260/230_), and RNA integrity numbers (RINs) are shown in Table 1 and Supplementary Table S1. The RINs of RNA from fresh blood samples (protocol P-Con; control samples) were above 7.0 (7.9 ± 0.7, mean ± standard deviation).

**Table 1:** Yield and quality of RNA extracted from peripheral whole blood samples using different conditions after short-term storage.

Sample group	Thawing condition	Purification kit	Duration of thawing (min)	A260/A280	A260/A230	Yield (μg/mL)	RIN
P-Con	Fresh blood	PAXgene		2.12 ± 0.31	1.76 ± 0.23	1.51 ± 0.63	7.9 ± 0.7
P-37	In water bath at 37 °C	PAXgene	5.1 ± 0.3	2.39 ± 0.56	1.18 ± 0.73	0.83 ± 0.35	5.1 ± 1.6
P-Al	On aluminum block At room temperature	PAXgene	17.8 ± 0.7	2.20 ± 0.04	1.76 ± 0.23	1.27 ± 0.45	6.6 ± 0.7
N-37	In water bath at 37 °C	NucleoSpin	4.9 ± 0.3	1.93 ± 0.23	1.43 ± 0.36	5.83 ± 2.31	5.4 ± 0.9
N-Al	On aluminum block At room temperature	NucleoSpin	17.7 ± 0.9	1.97 ± 0.07	1.49 ± 0.18	6.69 ± 2.04	5.8 ± 1.2
N-Ice	On ice	NucleoSpin	120	1.99 ± 0.16	1.49 ± 0.32	6.23 ± 2.29	5.8 ± 1.5

Samples were purified after short-term storage (less than 72 h, n = 7). Data are shown as mean ± standard deviation.

*A*_*260/280*_ absorbance ratio at 260 and 280 nm, *A*_*260/230*_ absorbance ratio at 260 and 230 nm, *RIN* RNA integrity number.

**Figure 2:**
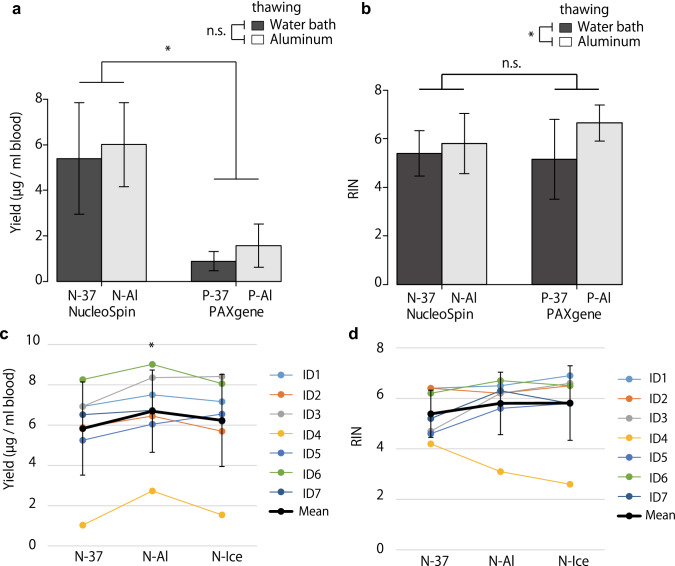
Comparison of yield and RNA integrity number (RIN) of RNA isolated from whole blood samples. (**a,b**) Comparison of (**a**) yield and (**b**) RIN of RNA among protocols P-37, P-Al, N-37, and N-Al. Data are shown as mean ± standard deviation (n = 7). *p < 0.05 (two-way analysis of variance) for (**a**) NucleoSpin versus PAXgene, or (**b**) thawing in a water bath versus thawing on an aluminum block. (**c,d**) Comparison of (**c**) yield and (**d**) RIN of RNA among protocols N-37, N-Al, and N-Ice. *p < 0.05 (repeated-measures analysis of variance with Bonferroni correction) for N-37 versus N-Al. *n.s.* not significant. The images were created using EZR software version 1.54 (http://www.jichi.ac.jp/saitama-sct/SaitamaHP.files/statmedEN.html), Microsoft Excel 2016 MSO (16.0.14131.20278) 32 bit (https://www.microsoft.com/) and Adobe Illustrator CS5 (https://www.adobe.com/).

The yields of RNA and RINs were compared among samples thawed in a water bath at 37 °C (protocols P-37 and N-37) and on an aluminum block at room temperature (protocols P-Al and N-Al), as well as among samples purified using the PAXgene kit (P-37 and P-Al) and the NucleoSpin kit (N-37 and N-Al). The RNA yields were significantly higher in the samples from the N-37 and N-Al groups than in those from the P-37 and P-Al groups (*p* = 1.4 × 10^−7^) (Fig. 2a). The RINs of the samples from the P-Al and N-Al groups, which were thawed on an aluminum block at room temperature, were significantly higher than those of the samples from the P-37 and N-37 groups, which were thawed in a water bath at 37 °C (*p* = 0.044) (Fig. 2b).

Next, the RNA yields and RINs were compared among three thawing conditions (N-37, N-Al, and N-Ice) using the NucleoSpin kit. The average yield of RNA from the N-Al samples, thawed on an aluminum block (6.69 ± 2.04 μg/mL of blood), was significantly higher than that from the N-37 samples, thawed in a water bath (5.83 ± 2.31 μg/mL of blood) (*p* = 0.014), and it was comparable to that from N-Ice samples, thawed on ice (6.23 ± 2.29 μg/mL of blood) (*p* = 0.26) (Fig. 2c). The RINs as well as A_260/280_ and A_260/230_ ratios were not significantly different among the three groups (Fig. 2d, Table 1). However, the N-37 group had three samples with RINs of 5 or less, whereas the N-Al and N-Ice groups had only one sample each (Fig. 2d, Supplementary Table S1, Fig. S1).

**Figure 3:**
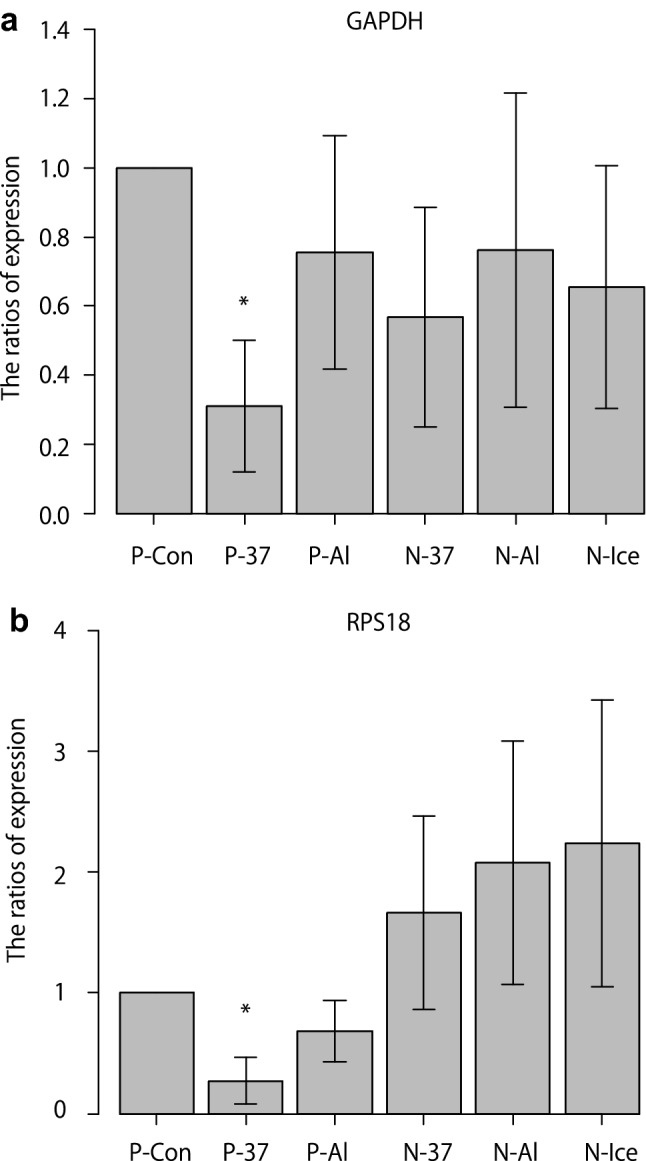
Comparison of *GAPDH* and *RPS18* expression in RNA isolated from whole blood samples using six different protocols. (**a**,**b**) Data are shown for each sample as mean ± standard deviation (n = 7). (**a**) *p < 0.05 for P-Con versus P-37 and P-37 versus P-Al (repeated-measures analysis of variance with Bonferroni correction). (**b**) *p < 0.05 for P-Con versus P-37, P-37 versus N-37, and P-37 versus N-Al (repeated-measures analysis of variance with Bonferroni correction). *GAPDH* glyceraldehyde 3-phosphate dehydrogenase, *RPS18* ribosomal protein S18. The images were created using EZR software version 1.54 (http://www.jichi.ac.jp/saitama-sct/SaitamaHP.files/statmedEN.html), Microsoft Excel 2016 MSO (16.0.14131.20278) 32 bit (https://www.microsoft.com/) and Adobe Illustrator CS5 (https://www.adobe.com/).

To further assess the quality of the RNA samples, glyceraldehyde 3-phosphate dehydrogenase (*GAPDH*, a housekeeping gene) mRNA was quantified using quantitative real-time polymerase chain reaction (qPCR). The mRNA expression was significantly lower in the P-37 group than in the P-Con (*p* = 0.001) and P-Al (*p* = 0.036) groups (Fig. 3a). Moreover, the expression in the N-37, N-Al, and N-Ice groups was not significantly different from that in the P-Con group. The mRNA expression of the ribosomal protein S18 (*RPS18*, another housekeeping gene) was significantly lower in the P-37 group than in the P-Con (*p* = 8.9 × 10^−4^), N-37 (*p* = 0.027), and N-Al (*p* = 0.033) groups (Fig. 3b).

### DNA quantity and quality

**Figure 4:**
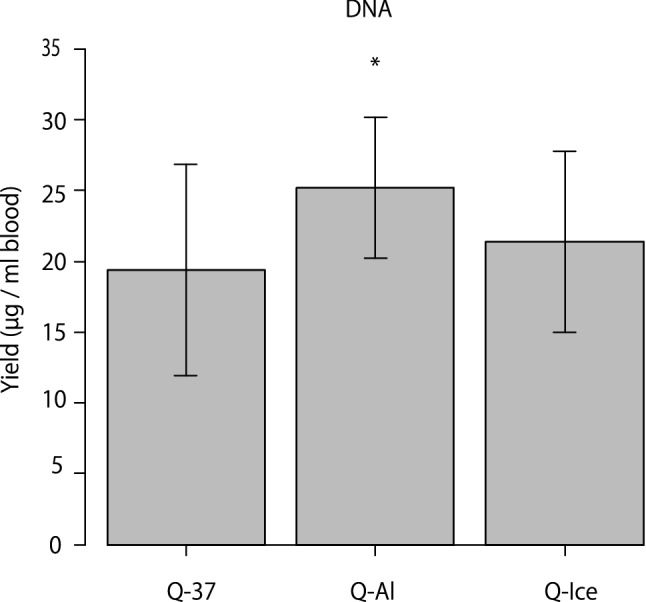
Comparison of the yield of DNA isolated from whole blood samples using protocols Q-37, Q-Al, and Q-Ice. Data are shown for each sample as mean ± standard deviation (n = 6). *p < 0.05 for Q-37 versus Q-Al and Q-Al versus Q-Ice (repeated-measures analysis of variance with Bonferroni correction). The image was created using EZR software version 1.54 (http://www.jichi.ac.jp/saitama-sct/SaitamaHP.files/statmedEN.html) and Adobe Illustrator CS5 (https://www.adobe.com/).

The yield and quality of DNA were compared among the three different thawing conditions (*n* = 6; Fig. 1). The DNA yield of the Q-Al group (aluminum block; 25.19 ± 4.99 μg/mL of blood) was significantly higher than that of the Q-37 (water bath; 19.40 ± 7.48 μg/mL of blood) (*p* = 0.023) and Q-Ice (on ice; 21.40 ± 6.37 μg/mL of blood) (*p* = 0.021) groups (Fig. 4, Table 2). Even though a statistically significant difference (*p* = 0.002) was observed between the Q-Al (1.91 ± 0.03) and Q-37 (1.90 ± 0.03) groups, it was not meaningful, as both A_260/280_ ratios were good. The DNA samples were electrophoresed, but no obvious smears were observed, suggesting that DNA degradation had not progressed (Supplementary Fig. S2).

**Table 2:** Yield and quality of DNA extracted from peripheral whole blood samples using different conditions after short-term storage.

Sample Group	Thawing condition	Purification kit	A260/A280	A260/A230	Yield (μg/mL)
Q-37	In water bath at 37 °C	QIAamp	1.90 ± 0.03	2.53 ± 0.11	19.40 ± 7.49
Q-Al	On aluminum block at room temperature	QIAamp	1.91 ± 0.03	2.47 ± 0.02	25.19 ± 4.99
Q-Ice	On ice	QIAamp	1.99 ± 0.21	2.53 ± 0.08	21.40 ± 6.37

Data are shown as mean ± standard deviation (n = 6).

*A*_*260/280*_ absorbance ratio at 260 and 280 nm, *A*_*260/230*_ absorbance ratio at 260 and 230 nm.

### RNA and DNA purification from frozen EDTA blood samples after long-term storage

Based on the data obtained, we concluded that the best protocols for RNA and DNA purification from frozen EDTA blood samples were N-Al and Q-Al (Fig. 1). Next, RNA and DNA were purified from frozen EDTA blood samples (Participants2, *n* = 30) after long-term storage (average duration: 6.1 ± 1.7 years; maximum duration: 8.5 years). The yield of RNA (9.07 ± 2.83 μg/mL of blood) was significantly higher than that after short-term storage (6.69 ± 2.04 μg/mL of blood) (p = 0.006), but it is difficult to make a simple comparison because the short-term storage samples were in 5 mL tubes and the long-term storage sample were in 7 mL tubes. The A_260/280_ and A_260/230_ ratios were 2.05 ± 0.03 and 1.65 ± 0.19, respectively, and the RIN and 28S/18S rRNA ratio were 5.9 ± 0.8 and 1.2 ± 0.3, respectively (Table 3, Supplementary Table S2). There was no correlation between the storage period and RIN or RNA yield (RIN: correlation coefficient =  − 0.115, p = 0.547; RNA yield: correlation coefficient =  − 0.01, p = 0.944). The yield of DNA was 18.16 ± 4.46 μg/mL of blood, with the A_260/280_ and A_260/230_ ratios of 1.92 ± 0.02 and 2.47 ± 0.08, respectively (Table 3, Supplementary Table S3).

**Table 3:** Yield and quality of RNA and DNA extracted from peripheral whole blood samples after long-term storage.

	A260/A280	A260/A230	Yield (μg/mL)	RIN	28S/18S
RNA	2.05 ± 0.03	1.65 ± 0.19	9.07 ± 2.83	5.9 ± 0.8	1.2 ± 0.3
DNA	1.92 ± 0.02	2.47 ± 0.08	18.15 ± 4.46		

Data are shown as mean ± standard deviation (n = 30).

*A*_*260/280*_ absorbance ratio at 260 and 280 nm, *A*_*260/230*_ absorbance ratio at 260 and 230 nm, *RIN* RNA integrity number, *28S/18S* 28S-to-18S rRNA ratio.

Finally, RNA sequencing (RNA-Seq) was performed using RNA from long-term storage samples (Participants2) and independent control RNA samples from fresh blood (Participants3, *n* = 10, Supplementary Table S4) and purified using the PAXgene kit. The coefficient of variation of the long-term storage samples for the expression of 23 housekeeping genes was approximately 30%, which was lower than that of the control (Supplementary Table S5). The inter-group variations in gene expression in the long-term storage samples were significantly lower for five of the 23 genes than those in the control samples by F-test (Supplementary Table S5).

## Discussion

In this study, RNA of high quantity and quality for expression analysis could be purified by appropriately thawing frozen whole blood samples and using the NucleoSpin RNA kit. Blood samples can be easily collected in a general clinic, requiring only EDTA collection tubes, and their simple storage in a deep freezer may enable the extraction of high-quality RNA and DNA even years later. We were able to extract high-quality RNA from whole blood samples frozen in EDTA tubes even after 8.5 years of storage. According to the NucleoSpin kit protocol provided by the supplier, it is recommended to add lysis buffer to the frozen sample and thaw it quickly in the buffer. However, whole blood in one EDTA tube needed to be thawed and aliquoted for DNA purification. We have shown that high-quality RNA can be extracted from blood samples thawed using an aluminum block. In this study, the NucleoSpin kit was used for long-term storage samples because a large amount of RNA was needed, but the PAXgene kit is also available for blood thawed using an aluminum block.

Freezing and thawing cycles are known to cause cell damage. Therefore, cryoprotectants, such as dimethyl sulfoxide (DMSO), are used when freezing and storing cells. Specifically, cryoprotectants are considered effective in preventing ice crystal formation in cells during freezing^12^. Rapid thawing of frozen cells containing DMSO has been reported to reduce cell death^12,15^. Therefore, cells are usually thawed rapidly at 37 °C in a water bath. However, without cryoprotectants, cell destruction is inevitable^21^, and RNA is degraded by leaked RNases, which are activated to a larger extent at 37 °C than at lower temperatures^22^. Nevertheless, thawing at a lower temperature requires a long time, which may result in RNA degradation. For example, it has been reported that thawing 2.7 mL of EDTA blood on ice takes 2 h to thaw completely^18^. The major aspect of our method is that we were able to reduce the thawing time to less than 20 min using aluminum blocks, although the samples were thawed at room temperature. Aluminum is known to have a high thermal conductivity^23^. It has been reported that an aluminum plate accelerates the thawing process of liver slices with improved cell viability^24^. Rapid thawing at lower temperatures prevents cell rupture and inhibits RNase activation, which suggests that RNA of good quality and quantity could be extracted.

In addition, our thawing method significantly increased the yield of DNA, which can be used to analyze epigenetics research involving methylation^25^, bacterial DNA^26^, and circulating DNA in plasma^27^. It has been reported that a single freeze–thaw step of blood reduced DNA yield by approximately 25%^20^. However, it was unclear why thawing with aluminum blocks would increase DNA yield, but we propose the following hypothesis. This research was inspired by the idea of how to cook a good steak. A previous study reported that steaks thawed at 20 °C for 20 min had significantly less thaw drip loss than those thawed at 4 °C for 20 h^28^. Furthermore, steaks thawed at 20 °C for 20 min had significantly less total moisture loss than those thawed at 39 °C for 11 min or at 4 °C for 20 h^28^. These findings suggest that moderate quick thawing decreases damage to frozen muscle structure. A similar phenomenon might occur in frozen blood. The decrease in cell damage may have affected the yield of DNA.

Our methodology may offer various benefits in clinical studies. First, cryopreserved whole blood samples can be used for both RNA and DNA analyses. Second, the RNA purification protocol can be inexpensively modified with no special equipment other than an aluminum block for thawing. The cost of a single aluminum block is less than 1 US dollar, and it can be used for blood collection tubes of any shape and size. Third, the number of storage tubes can be reduced because DNA and RNA can be purified from a single EDTA tube of whole blood. Finally, the viability of other stored cells may be increased by further developing an appropriate thawing method.

However, there are some limitations to this study. First, cryopreservation conditions were not studied. Previous investigations have reported that the freezing method has a greater impact on cell survival and gene expression than the thawing method^7,14,15,29^. Second, we did not examine materials other than aluminum for thawing. For example, copper and silver are known to have higher thermal conductivity than aluminum and may be more efficient than aluminum for thawing. Third, only some RNA purification products were tested. We preliminarily tried to purify RNA from frozen and thawed EDTA blood using a TRIzol kit; however, the RNA was degraded (data not shown). Indeed, a previous study has reported that the NucleoSpin kit is the most suitable for RNA purification from frozen blood^17^. Finally, a control using fresh blood and Nucleospin was not set up due to limited samples. Although there were no statistically significant differences, *RPS18* tends to be higher in NucleoSpin-purified samples than in PAXgene-purified samples. We checked the size of the *GAPDH* and *RPS18* amplicons, and found a single band of the expected size, with no unspecific amplification (Supplementary Figs. S3, S4). The difference may be largely due to the differences in purification kits, because such differences were observed in the results of samples thawed using an aluminum block (Fig. 3b, P-Al vs. N-Al). Similarly, in RNA-Seq, differences in the expression of housekeeping genes were observed between RNA from long-term storage cryopreserved samples and that from fresh blood (Supplementary Table S5). The reason is not clear, but it may be due to the differences in the purification kits or in the uniformity of the participants.

In summary, the thawing method using an aluminum block allowed the extraction of good-quality RNA and DNA from cryopreserved EDTA whole blood samples using commercial kits. This finding could promote the efficient use of blood stored in biobanks for future research on biomarkers and the pathogenesis of several diseases, such as blood disorders. Further investigations on the appropriate cooling methods and cytoprotective agents may lead to more convenient methods of cryopreservation of whole blood samples.

## Methods

### Subjects

#### Participants1

Eight participants were recruited at the Yamaguchi University Hospital using community advertisements for the depression stratification project (approval number H30-172) between 2019 and 2020. RNA and DNA were extracted from five individuals; from two individuals, only RNA was extracted and from one individual, only DNA was extracted.

#### Participants2

Thirty participants were recruited at the Hiroshima University Hospital and collaborating local clinics between 2012 and 2018 for the depression biomarker project (approval number H-35).

#### Participants3

Ten participants were recruited at Tokushima University Hospital (approval number R3-24).

The demographic data of all the participants are shown in Supplementary Table S6. The Institutional Review Board of the Yamaguchi University Hospital, the Ethics Committee of Hiroshima University, and the Institutional Ethics Committee of the University of Tokushima Graduate School approved this study. All subjects provided informed consent for participation. This study was carried out in accordance with the latest version of the Declaration of Helsinki.

### RNA isolation from human blood samples

The RNA purification protocol is summarized in Fig. 1. At the Yamaguchi University Hospital, venous blood samples (5 mL each) were collected from the participants into EDTA-Na_2_ tubes (Terumo Corporation, Tokyo, Japan) or PAXgene tubes (Becton, Dickinson and Company, Franklin Lakes, NJ, USA). RNA isolation from samples filled in PAXgene tubes was performed using the PAXgene Blood RNA Kit (Qiagen, Venlo, Netherlands) according to the manufacturer’s protocol. The filled EDTA tubes were immediately placed in a − 20 °C freezer and transferred to a − 80 °C deep freezer the next day. RNA was purified after 48–72 h of storage. At the Hiroshima University Hospital and collaborating local clinics, venous blood samples (7 mL from each participant) were collected into EDTA-Na_2_ tubes and stored in a − 80 °C deep freezer for several years. Detailed conditions until cryopreservation were not recorded. At the Tokushima University Hospital, PAXgene Blood RNA tubes (Qiagen) and PAXgene Blood RNA Kits (Qiagen) were used according to the manufacturer’s recommendations to extract the total RNA from whole blood samples.

**Figure 5:**
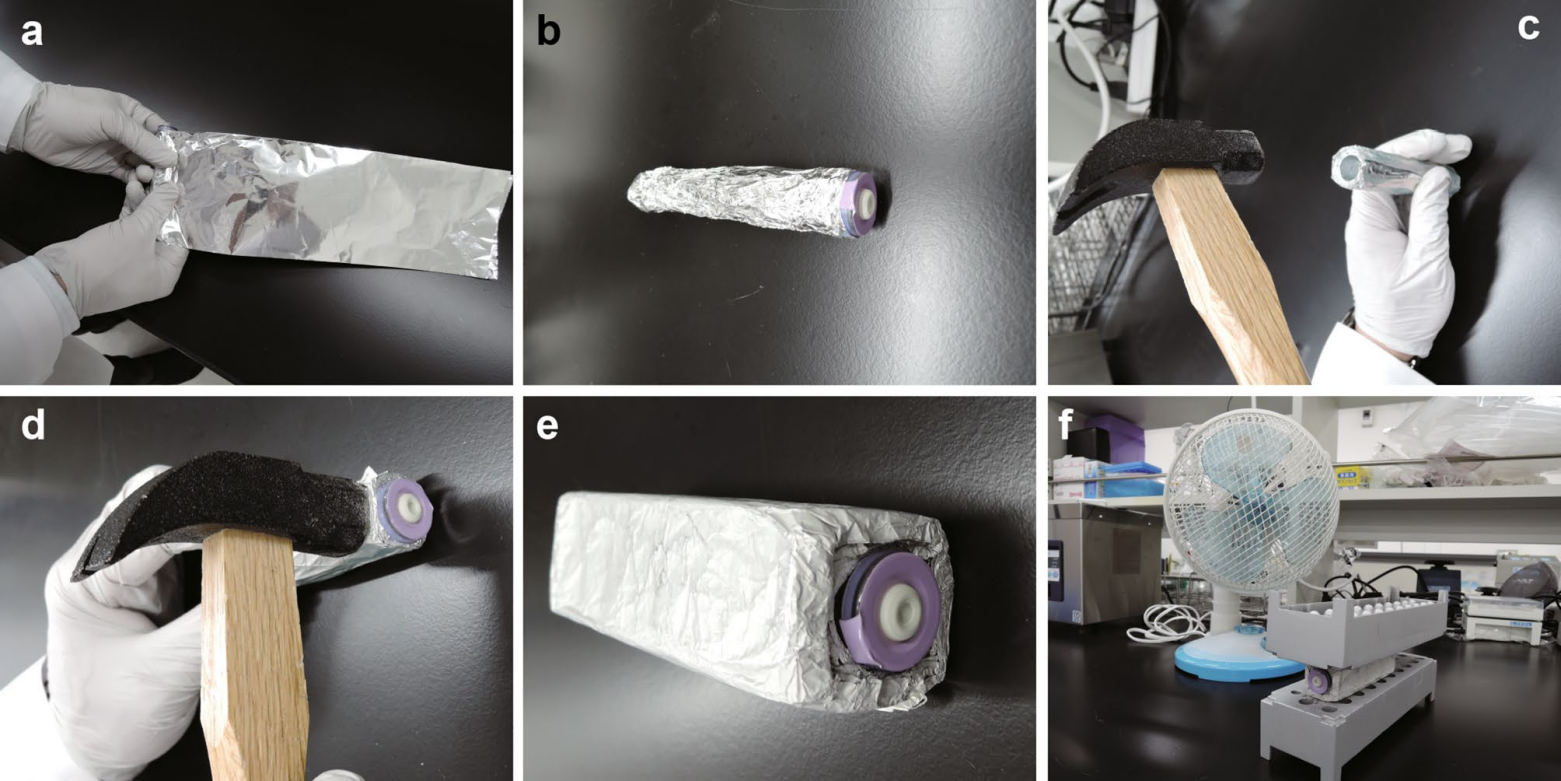
Procedure for thawing frozen whole blood samples stored in ethylenediaminetetraacetic acid (EDTA) tubes. (**a**) Wrap aluminum foil tightly around the EDTA tube. (**b**) Repeat the wrapping process until the aluminum foil does not lose its shape when the EDTA tube is pulled out. (**c**) Beat the top and bottom of the aluminum with a hammer. (**d**) Beat the side of the aluminum with a hammer. (**e**) Repeat steps (**a–d**) until the thickness of the aluminum block is around 1 cm. (**f**) Place the EDTA tube containing the frozen whole blood sample in an aluminum block, sandwich it between aluminum racks for microtubes, and blow it with a fan. The images were created using Adobe Illustrator CS5 (https://www.adobe.com/).

The frozen samples were thawed under the following conditions: in a water bath at 37 °C for approximately 5 min, on an aluminum block at room temperature (23 °C) for approximately 18 min, or on ice for 2 h. Original aluminum blocks were prepared by wrapping an EDTA tube with aluminum foil and beating it with a hammer (see Fig. 5 for detailed instructions on how to thaw using an aluminum block). The thawed blood samples were immediately transferred into PAXgene tubes or to the lysis buffer from the NucleoSpin RNA blood kit (Takara Bio, Kusatsu, Japan). RNA isolation was performed according to the manufacturer’s protocol. The RNA quantity and quality were determined using a NanoDrop One spectrophotometer (Thermo Fisher Scientific, Waltham, MA, USA). The RINs were determined using an Agilent Bioanalyzer with the Agilent RNA 6000 nano or pico kit (Agilent Technologies, Santa Clara, CA, USA) according to the manufacturer’s protocol. The 28S/18S rRNA ratios were calculated using the area of each peak in the electropherogram.

Genomic DNA was purified from the thawed whole blood samples using the QIAamp DNA blood midi kit (Qiagen) according to the manufacturer’s instructions. The DNA quality was determined using a NanoDrop One spectrophotometer.

### qPCR

cDNA synthesis and qPCR were performed using a previously reported method^30^, with slight modifications. Briefly, cDNA was synthesized using the PrimeScript RT reagent kit (Takara Bio) with oligo(dT) primers and 1 μg of total RNA. The synthesized cDNA was mixed with SYBR Premix Ex Taq II (Takara Bio) and specific primers. Amplification was performed for 50 cycles, each at 95 °C for 15 s and 60 °C for 1 min, using a StepOnePlus real-time PCR system (Thermo Fisher Scientific). The list of primers used is presented in Supplementary Table S7. All measurements were performed in duplicate. For quantifying the levels of *GAPDH* and *RPS18*, a calibration curve was generated using 10, 5, 2.5, 1.25, 0.625, and 0.3125 fM synthetic DNA, which included the target sequence (*GAPDH*, GenScript Japan, Tokyo, Japan; *RPS18*, Integrated DNA Technologies, Coralville, IA, USA). The list of synthetic DNA sequences used is provided in Supplementary Table S8. For gel images, amplification was performed for 35 cycles, each at 95 °C for 15 s and 60 °C for 1 min, using an Applied Biosystems 2720 Thermal Cycler (Thermo Fisher Scientific).

### RNA sequencing

Sequencing libraries were constructed from 100 ng (for Participants2) or 300 ng (for Participants3) of RNA using the TruSeq Stranded Total RNA with Ribo-Zero Gold LT sample prep kit (Illumina, San Diego, CA, USA) according to the manufacturer’s instructions. The libraries were pooled after quantification by Bioanalyzer analysis and fluorometry using the Qubit dsDNA HS assay kit and a Qubit 2.0 fluorometer (Thermo Fisher Scientific). Sequencing of paired-end fragments (75 bp × 2) was conducted on the NextSeq 500 sequencing platform (Illumina) to a depth of 13 to 35 (average: 21) million fragments.

Data for each sample were separated to generate FASTQ files. Next-generation sequencing data were cleaned using cutadapt (version 1.8.3)^31^ and cmpfastq_pe.pl (http://compbio.brc.iop.kcl.ac.uk/software/cmpfastq_pe.php). After a quality control step, the filtered short reads were mapped to the reference genome (hg38) using STAR (version 2.5.1b)^32^. Strand-specific transcripts per million were obtained from each sample using RSEM (version 1.3.3.)^33^.

### Statistical analysis

RINs, mRNA expression, A_260/280_ ratios, and RNA and DNA yields were analyzed using a two-way analysis of variance or repeated-measures analysis of variance with Bonferroni correction with EZR software version 1.54 (http://www.jichi.ac.jp/saitama-sct/SaitamaHP.files/statmedEN.html)^34^. The housekeeping gene expression in RNA-Seq was analyzed using F-test with Bonferroni correction using EZR software version 1.54. Results with *P* ≤ 0.05 were considered statistically significant. A statistical summary is shown in Supplementary Table S9.

## Supplementary Information

Supplementary Information.

## Data Availability

All data are available within this published article and its Supplementary Information.
