# Supplementary material for: Optimized protocol for the extraction of RNA and DNA from frozen whole blood sample stored in a single EDTA tube
Source: Sci Rep. 2021 Aug 23;11:17075. doi: 10.1038/s41598-021-96567-2 (PMC8382694; doi:10.1038/s41598-021-96567-2)

Optimized protocol for the extraction of RNA and DNA from frozen whole blood sample stored in a single EDTA tube

Hirotaka Yamagata[1,2]*, Ayumi Kobayashi[1], Ryouichi Tsunedomi[3], Tomoe Seki[1,2], Masaaki Kobayashi[1], Kosuke Hagiwara[1], Chong Chen[1], Shusaku Uchida[2,4], Go Okada[5], Manabu Fuchikami[5], Toshiharu Kamishikiryo[5], Jun-ichi Iga[6], Shusuke Numata[7], Makoto Kinoshita[7], Takahiro A. Kato[8], Ryota Hashimoto[9], Hiroaki Nagano[3], Yasumasa Okamoto[5], Shuichi Ueno[6], Tetsuro Ohmori[7], & Shin Nakagawa[1]

1 Division of Neuropsychiatry, Department of Neuroscience, Yamaguchi University Graduate School of Medicine, 1-1-1 Minami-kogushi, Ube, Yamaguchi 755-8505, Japan.

2 Core Research for Evolutional Science and Technology (CREST), Japan Science and Technology Agency (JST), 4-1-8 Honcho, Kawaguchi, Saitama 332-0012, Japan.

3 Department of Gastroenterological, Breast and Endocrine Surgery, Yamaguchi University Graduate School of Medicine, 1-1-1 Minami-kogushi, Ube, Yamaguchi 755-8505, Japan.

4 SK Project, Medical Innovation Center, Kyoto University Graduate School of Medicine, 53 Shogoin-Kawahara-cho, Sakyo-ku, Kyoto 606-8507, Japan.

5 Department of Psychiatry and Neurosciences, Graduate School of Biomedical Sciences, Hiroshima University, 1-2-3 Kasumi, Minami-ku, Hiroshima, 734-8551, Japan.

6 Department of Neuropsychiatry, Molecules and Function, Ehime University Graduate School of Medicine, Shitsukawa, Toon, Ehime, 791-0295, Japan.

7 Department of Psychiatry, Graduate School of Biomedical Sciences, Tokushima University, 3-18-5 Kuramoto-cho, Tokushima, 770-8503, Japan.

8 Department of Neuropsychiatry, Graduate School of Medical Sciences, Kyushu University, 3-1-1 Maidashi Higashi-Ku, Fukuoka, 812-8582, Japan.

9 Department of Pathology of Mental Diseases, National Institute of Mental Health, National Center of Neurology and Psychiatry, 4-1-1 Ogawa-Higashi, Kodaira, Tokyo 187-8553, Japan.

*Correspondence to:

Hirotaka Yamagata, M.D., Ph.D.

Division of Neuropsychiatry, Department of Neuroscience, Yamaguchi University Graduate School of Medicine, 1-1-1 Minami-kogushi, Ube, Yamaguchi 755-8505, Japan

Tel: +81 836 22 2255; Fax: +81 836 22 2253; e-mail: gata@yamaguchi-u.ac.jp

Supplementary information

Supplementary Table S1. Characteristics of RNA purified from peripheral whole blood sample under different conditions after short-term storage (Participants1). Data are shown as mean ± standard deviation. A260/280, absorbance ratio at 260 and 280 nm; A260/230, absorbance ratio at 260 and 230 nm; RIN, RNA integrity number; 28S/18S, 28S-to-18S rRNA ratio.

Supplementary Table S2. Characteristics of RNA purified from peripheral whole blood sample after long-term storage (Participants2). A260/280, absorbance ratio at 260 and 280 nm; A260/230, absorbance ratio at 260 and 230 nm; RIN, RNA integrity number; 28S/18S, 28S-to-18S rRNA ratio.

Supplementary Table S3. Characteristics of DNA purified from peripheral whole blood sample after long-term storage (Participants2). A260/280, absorbance ratio at 260 and 280 nm; A260/230, absorbance ratio at 260 and 230 nm.

Supplementary Table S4. Characteristics of RNA purified from peripheral whole blood sample (Participants3). RIN, RNA integrity number.

Supplementary Table S5. Expression of housekeeping genes in long-term storage samples (Participants2, n = 30) and control samples of fresh blood (Participants3, n = 10). SD, standard deviation; CV, coefficient of variation.

Supplementary Table S6. Demographic characteristics of the participants. Age is shown as mean ± standard deviation.

Supplementary Table S7. Primer sequences used in this study and amplicon lengths.

Supplementary Table S8. Sequences of synthetic DNA.

Supplementary Table S9. Summary of statistical analyses.

Supplementary Fig. S1 Typical electropherograms of purified RNA. Thawing in a water bath increased the RNA degradation in samples P-37 and N-37 of participants 3 and 5.

Supplementary Fig. S2 Gel images of DNA. Agarose gel (1%) imaging was performed using ATTO printgraph and ATTO imageSaver AE-6905C. Five hundred nano grams of DNA was loaded in each lane. Five microliters of GeneRuler 100-bp Plus DNA Ladder (Thermo Fisher Scientific) was used for loading controls and markers.

Supplementary Fig. S3 Gel images of PCR products of GAPDH. Agarose gel (1.2%) imaging was performed using ATTO printgraph and ATTO imageSaver AE-6905C. Three microliters of GeneRuler 100-bp Plus DNA Ladder (Thermo Fisher Scientific) was used for loading controls and markers.

Supplementary Fig. S4 Gel images of PCR products of RPS18. Agarose gel (1.2%) imaging was performed using ATTO printgraph and ATTO imageSaver AE-6905C. Three microliters of GeneRuler 100-bp Plus DNA Ladder (Thermo Fisher Scientific) was used for loading controls and markers.

Table S1

protocol	ID	time for thawing (minutes)	A260/A280	A260/A230	yeild (ug/mL) by A260	yeild (ug/mL) by Qubit	RIN	28S/18S
P-Con	1	—	2.09	1.87	2.03	2.13	8.4	1.3
	2	—	2.14	1.71	0.81	0.89	7.9	1.9
	3	—	2.14	1.51	1.91	2.00	6.9	1.8
	4	—	2.16	1.58	0.84	0.98	7.7	1.7
	5	—	2.07	2.08	2.11	1.73	7.3	2.0
	6	—	2.12	2.02	2.02	2.22	8	1.9
	7	—	2.11	1.56	0.86	0.94	9.2	1.6
P-37	1	4.7	2.17	1.76	1.36	1.48	6.7	1.5
	2	4.7	2.22	1.52	0.90	1.05	6.4	1.4
	3	5.2	2.42	0.24	0.91	0.92	4.2	1.0
	4	5.2	3.63	0.07	0.27	0.16	2.1	0.4
	5	5.3	2.05	1.48	0.73	0.79	4.5	1.1
	6	5.3	2.11	1.89	1.08	1.16	6	1.3
	7	5.3	2.15	1.32	0.60	0.65	6.1	1.0
P-Al	1	18.0	2.2	1.44	1.22	1.34	7	1.5
	2	18.0	2.17	1.89	1.68	1.77	7.5	1.8
	3	16.8	2.16	1.95	2.02	3.59	7.2	0.9
	4	16.8	2.22	1.45	0.71	0.77	5.3	1.4
	5	18.3	2.22	1.75	1.24	1.35	6.6	1.1
	6	18.3	2.18	1.94	1.19	1.18	6.8	1.1
	7	18.3	2.27	1.95	0.85	0.95	6.1	1.3
N-37	1	4.5	2.04	1.64	6.93	6.30	6.4	0.8
	2	4.5	2.01	1.59	5.88	5.40	6.4	0.8
	3	5.3	2	1.62	6.93	7.06	4.7	0.8
	4	5.3	1.42	0.69	1.04	0.43	4.2	1.6
	5	4.8	2	1.39	5.25	4.73	4.6	0.9
	6	4.8	2.08	1.79	8.26	8.04	6.2	0.8
	7	4.8	1.98	1.32	6.52	5.82	5.2	0.7
N-Al	1	16.5	2.01	1.71	7.50	6.47	6.5	0.9
	2	16.5	2.02	1.5	6.45	5.54	6.2	0.9
	3	17.7	2.01	1.72	8.36	8.18	6.2	0.6
	4	17.7	1.81	1.23	2.73	2.60	3.1	1.0
	5	18.5	2	1.44	6.05	5.60	5.6	1.0
	6	18.5	2	1.47	9.02	7.87	6.7	0.9
	7	18.5	1.96	1.36	6.73	5.80	6.3	0.6
N-Ice	1	120	2.05	1.68	7.17	6.58	6.9	1.3
	2	120	2.03	1.5	5.70	5.35	6.5	0.9
	3	120	2.04	1.7	8.41	8.12	6.6	1.1
	4	120	1.64	0.82	1.54	0.87	2.6	1.8
	5	120	2.02	1.42	6.55	5.54	5.8	1.0
	6	120	2.11	1.79	8.06	7.36	6.5	0.7
	7	120	2.05	1.54	6.20	5.32	5.8	0.9

Table S2

ID	sex	age	A260/A280	A260/A230	Yield (μg/ml)	RIN	28S/18S	Preservation period (years)
Dep007	F	25	2.01	1.49	6.67	5.9	1	8.5
Dep008	F	28	2.09	1.67	8.43	5.8	1.3	8.5
Dep029	M	39	2.08	1.62	9.58	5.4	1.4	8.2
Dep053	F	36	2.10	1.45	6.46	5.9	1	7.9
Dep060	F	39	2.01	1.58	6.95	5.6	1.4	7.8
Dep064	M	51	2.05	1.47	5.13	5.1	1.3	7.7
Dep077	M	44	2.08	1.94	11.90	6	0.9	7.6
Dep085	F	42	2.06	1.33	8.50	5.8	1.5	7.5
Dep093	M	41	2.10	1.83	13.96	6.4	1.7	7.4
Dep103	F	28	2.02	1.72	9.50	6.6	1.1	7.3
Dep130	M	47	2.05	1.77	8.00	5.3	1.4	6.7
Dep135	F	46	2.09	1.86	10.60	4.7	0.9	6.6
Dep137	M	33	2.06	1.96	16.98	4.7	0.7	6.5
Dep140	F	33	2.04	1.76	10.19	7	1.5	6.5
Dep142	M	55	2.05	1.48	9.33	7.1	1.2	6.5
Dep147	F	27	2.05	1.65	6.82	5.6	1.1	6.4
Dep153	F	41	2.03	1.70	8.71	7	1.3	6.3
Dep166	F	41	2.07	1.22	6.98	6.7	0.6	6.0
Dep169	F	27	2.06	1.40	5.42	5.3	1	6.0
Dep172	M	29	2.10	1.92	15.59	5	0.9	5.9
Dep173	M	43	2.06	1.60	6.73	5.5	1	5.8
Dep176	M	40	2.06	1.86	10.46	6.5	1	5.8
Dep180	M	28	2.02	1.55	6.35	7.1	1.8	5.6
Dep183	F	43	2.05	1.78	10.64	4.9	0.9	5.5
Dep211	F	39	2.05	1.45	7.84	5.7	1.1	5.1
Dep215	M	34	2.04	1.76	10.42	7.4	1.3	5.0
FD010	F	39	2.01	1.57	6.16	7.3	1.8	2.6
FD014	M	25	2.10	1.61	10.97	5.5	1	2.5
FD016	F	33	2.04	1.75	8.87	6.9	1.3	2.5
FD020	M	31	2.04	1.71	7.87	4.7	1	2.4

Table S3

ID	sex	age	A260/A280	A260/A230	Yield (μg/ml)	Preservation period (years)
Dep007	F	25	1.94	2.62	14.8	8.5
Dep008	F	28	1.93	2.40	18.7	8.5
Dep029	M	39	1.93	2.45	24.0	8.2
Dep053	F	36	1.92	2.37	15.9	7.9
Dep060	F	39	1.94	2.47	13.8	7.8
Dep064	M	51	1.91	2.45	15.3	7.7
Dep077	M	44	1.96	2.65	15.1	7.6
Dep085	F	42	1.91	2.41	21.6	7.5
Dep093	M	41	1.93	2.45	15.3	7.4
Dep103	F	28	1.94	2.54	18.5	7.3
Dep130	M	47	1.95	2.64	22.7	6.7
Dep135	F	46	1.91	2.38	16.6	6.6
Dep137	M	33	1.94	2.53	17.1	6.5
Dep140	F	33	1.91	2.36	25.6	6.5
Dep142	M	55	1.93	2.45	15.4	6.5
Dep147	F	27	1.93	2.43	16.6	6.4
Dep153	F	41	1.91	2.34	25.9	6.3
Dep166	F	41	1.91	2.45	18.9	6.0
Dep169	F	27	1.90	2.39	11.8	6.0
Dep172	M	29	1.93	2.49	13.8	5.9
Dep173	M	43	1.90	2.49	17.3	5.8
Dep176	M	40	1.90	2.41	25.1	5.8
Dep180	M	28	1.91	2.47	14.7	5.6
Dep183	F	43	1.91	2.38	22.0	5.5
Dep211	F	39	1.90	2.45	22.2	5.1
Dep215	M	34	1.92	2.54	12.2	5.0
FD010	F	39	1.94	2.49	13.8	2.6
FD014	M	25	1.93	2.54	20.7	2.5
FD016	F	33	1.96	2.51	12.6	2.5
FD020	M	31	1.94	2.43	26.8	2.4

Table S4

ID	sex	age	Concentration (μg/mL)	RIN
t1	F	25	100.0	8.5
t2	M	28	79.8	8.7
t3	M	39	92.0	8.4
t4	M	36	70.4	8.7
t5	F	39	94.2	8.6
t6	M	51	108.6	8.6
t7	F	44	83.6	8.8
t8	F	42	50.2	8.3
t9	F	41	43.0	7.8
t10	M	28	80.4	7.2

Table S5

genes	Participants2			Participants3			corrected p-values of F-test
	Mean (TPM)	SD	CV	Mean (TPM)	SD	CV	
ACTB	2530.19	596.71	0.24	774.78	596.57	0.77	2.0.E-05
ALAS1	9.34	2.80	0.30	10.83	4.42	0.41	1.000
ATP5F1	30.77	9.44	0.31	32.98	12.41	0.38	1.000
B2M	4287.65	1188.12	0.28	4330.86	1372.86	0.32	1.000
G6PD	25.33	6.37	0.25	11.81	7.29	0.62	4.4.E-03
GAPDH	345.33	91.27	0.26	288.59	122.27	0.42	1.000
GUSB	11.15	2.96	0.27	6.62	2.85	0.43	1.000
HMBS	6.24	1.06	0.17	9.53	2.11	0.22	1.000
HPRT1	6.58	2.01	0.31	5.20	2.64	0.51	0.838
IPO8	11.89	3.60	0.30	10.00	4.53	0.45	1.000
PGK1	81.94	22.28	0.27	136.64	55.84	0.41	1.000
POLR2A	22.11	7.31	0.33	7.23	6.65	0.92	4.7.E-04
PPIA	68.97	21.35	0.31	58.57	21.25	0.36	1.000
RPL13A	898.94	240.66	0.27	535.97	184.30	0.34	1.000
RPLP0	370.98	103.78	0.28	319.62	99.42	0.31	1.000
RPLP1	487.64	157.09	0.32	101.21	106.98	1.06	1.8.E-05
RPLP2	1190.20	257.46	0.22	816.98	280.86	0.34	1.000
RPS18	1254.58	365.46	0.29	550.45	186.51	0.34	1.000
SDHA	17.93	4.69	0.26	14.82	8.96	0.60	0.012
TBP	5.66	1.85	0.33	3.72	1.86	0.50	1.000
TFRC	50.36	10.93	0.22	13.30	3.85	0.29	1.000
UBC	101.02	29.35	0.29	184.46	63.82	0.35	1.000
YWHAZ	249.88	76.32	0.31	139.48	65.28	0.47	1.000

Table S6

	number	sex (Male/Female)	age
Participants1 for RNA<br>(short-term strorage)	n = 7	3/4	38.6 ± 8.3
Participants1 for DNA<br>(short-term strorage)	n = 6	2/4	37.5 ± 8.5
Participants2<br>(long-term strorage)	n = 30	14/16	36.9 ± 7.9
Participants3<br>(Fresh blood)	n = 10	5/5	53.7 ± 8.9

Table S7

GAPDH	Forward	CAGCCTCAAGATCATCAGCA
	Reverse	TGTGGTCATGAGTCCTTCCA
	amplicon lengths	106 base pairs
RPS18	Forward	CACTTCTGGGGCCTTCGTG
	Reverse	TTCTTGGACACACCCACGGT
	amplicon lengths	80 base pairs

Table S8

GAPDH	TGTTCGTCATGGGTGTGAACCATGAGAAGTATGACAACAGCCTCAAGATCATCAGCAATGC<br>CTCCTGCACCACCAACTGCTTAGCACCCCTGGCCAAGGTCATCCATGACAACTTTGGTATC<br>GTGGAAGGACTCATGACCACAGTCCATGCCATCACTGCCACCCAGAAGACTGTGGATGGC<br>CCCTCCGGGAAACTGTGGCGTGATGGCCGCGGGGCTCTCCAGAACATCATCCCTGCCTCT<br>ACTGGCGC
RPS18	ACAGACAGAAGGATGTAAAGGATGGAAAATACAGCCAGGTCCTAGCCAATGGTCTGGACAA<br>CAAGCTCCGTGAAGACCTGGAGCGACTGAAGAAGATTCGGGCCCATAGAGGGCTGCGTCA<br>CTTCTGGGGCCTTCGTGTCCGAGGCCAGCACACCAAGACCACTGGCCGCCGTGGCCGCAC<br>CGTGGGTGTGTCCAAGAAGAAATAAGTCTGTAGGCCTTGTCTGTTAATAAATAGTTTATAT<br>ACCTATGG

Table S9

Measurement	Statistical test	Comparison	Statisitcs	P value	Figures and Tables
Yield of RNA	2-way ANOVA	Factor1: thawing	F(1,24) = 1.11	0.302	Fig. 2a
		Factor2: kit	F(1,24) = 53.75	1.40E-07	
		Interaction (F1 x F2)	F(1,24) = 0.003	0.957	
RIN	2-way ANOVA	Factor1: thawing	F(1,24) = 4.53	0.044	Fig. 2b
		Factor2: kit	F(1,24) = 0.45	0.511	
		Interaction (F1 x F2)	F(1,24) = 1.46	0.239	
Yield of RNA	Repeated measures ANOVA		F(2,12) = 6.76	0.011	Fig. 2c
	post-hoc test	N-37 vs N-Al		0.014	
		N-37 vs N-Ice		0.588	
		N-Al vs N-Ice		0.256	
RIN	Repeated measures ANOVA		F(2,12) = 1.16	0.107	Fig. 2d
	post-hoc test	N-37 vs N-Al		0.310	
		N-37 vs N-Ice		1	
		N-Al vs N-Ice		0.390	

GAPDH	Repeated measures ANOVA		F(5,30) = 8.57	3.90E-05	Fig. 3a
	post-hoc test	P-Con vs P-37		0.001	
		P-Con vs P-Al		1	
		P-Con vs N-37		0.171	
		P-Con vs N-Al		1	
		P-Con vs N-Ice		0.596	
		P-37 vs P-Al		0.036	
		P-37 vs N-37		0.495	
		P-37 vs N-Al		0.495	
		P-37 vs N-Ice		0.391	
		P-Al vs N-37		0.484	
		P-Al vs N-Al		1	
		P-Al vs N-Ice		1	
		N-37 vs N-Al		0.807	
		N-37 vs N-Ice		1	
		N-Al vs N-Ice		1	
RPS18	Repeated measures ANOVA		F(5,30) = 14.91	2.30E-07	Fig. 3b
	post-hoc test	P-Con vs P-37		8.90E-04	
		P-Con vs P-Al		0.260	
		P-Con vs N-37		1	
		P-Con vs N-Al		0.448	
		P-Con vs N-Ice		0.495	
		P-37 vs P-Al		0.068	
		P-37 vs N-37		0.027	
		P-37 vs N-Al		0.033	
		P-37 vs N-Ice		0.052	
		P-Al vs N-37		0.156	
		P-Al vs N-Al		0.066	
		P-Al vs N-Ice		0.122	
		N-37 vs N-Al		1	
		N-37 vs N-Ice		0.540	
		N-Al vs N-Ice		1	
Yield of DNA	Repeated measures ANOVA		F(2,10) = 14.20	0.001	Fig. 4
	post-hoc test	Q-37 vs Q-Al		0.023	
		Q-37 vs Q-Ice		0.353	
		Q-Al vs Q-Ice		0.021	

ACTB	F-test	Participants2 vs Participants3	F(9,29) = 10.66	2.04E-05	Table S4
ALAS1			F(9,29) = 1.85	1	
ATP5F1			F(9,29) = 1.50	1	
B2M			F(9,29) = 1.31	1	
G6PD			F(9,29) = 6.02	4.45.E-03	
GAPDH			F(9,29) = 2.57	1	
GUSB			F(9,29) = 2.62	1	
HMBS			F(9,29) = 1.70	1	
HPRT1			F(9,29) = 2.76	0.838	
IPO8			F(9,29) = 2.24	1	
PGK1			F(9,29) = 2.26	1	
POLR2A			F(9,29) = 7.75	4.73.E-04	
PPIA			F(9,29) = 1.37	1	
RPL13A			F(9,29) = 1.65	1	
RPLP0			F(9,29) = 1.24	1	
RPLP1			F(9,29) = 10.77	1.84E-05	
RPLP2			F(9,29) = 2.53	1	
RPS18			F(9,29) = 1.36	1	
SDHA			F(9,29) = 5.34	0.012	
TBP			F(9,29) = 2.32	1	
TFRC			F(9,29) = 1.78	1	
UBC			F(9,29) = 1.42	1	
YWHAZ			F(9,29) = 2.35	1	

Figure S1

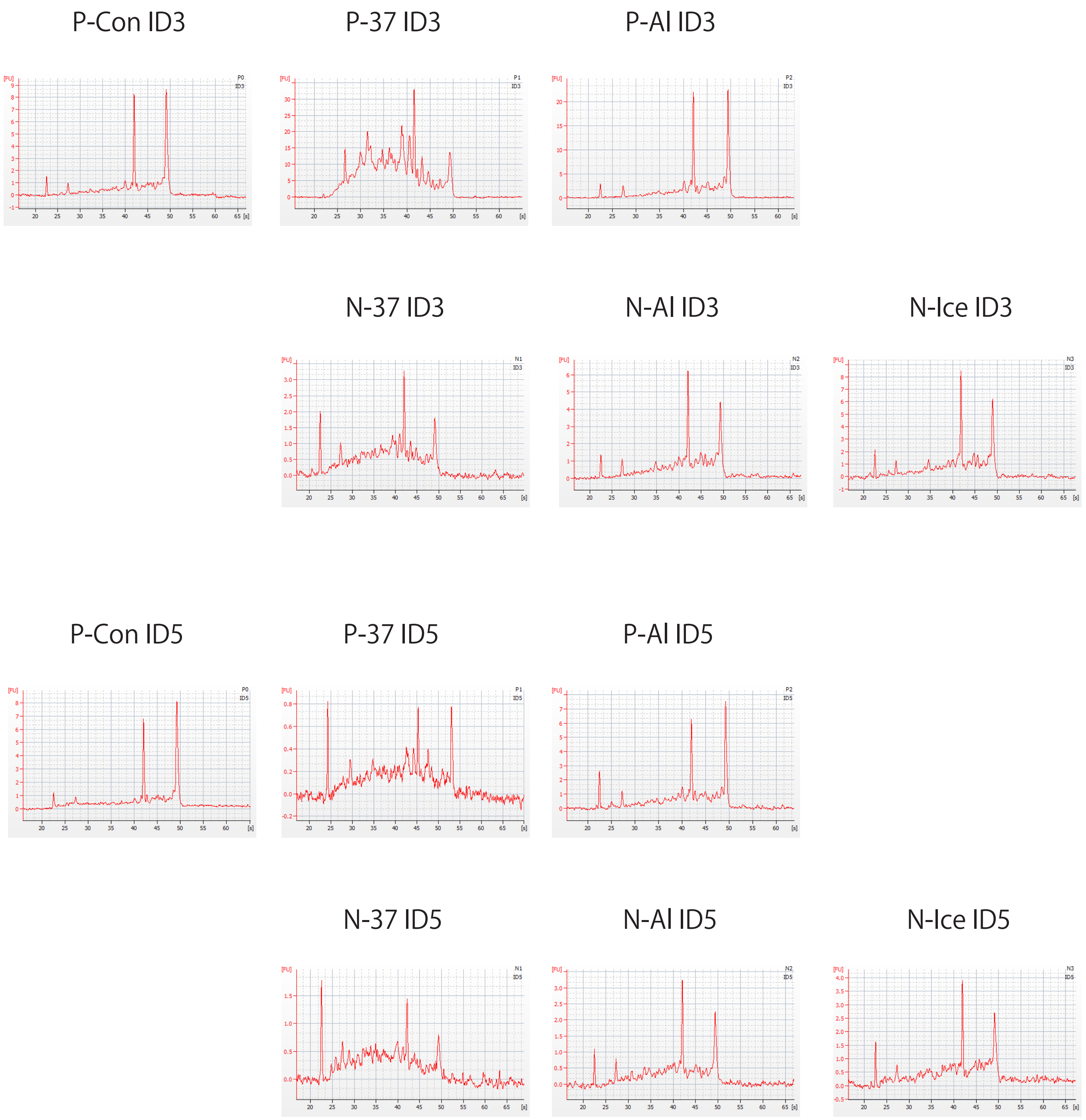
P-Con ID3
P-37 ID3
P-Al ID3
N-37 ID3
N-Al ID3
N-Ice ID3
P-Con ID5
P-37 ID5
P-Al ID5
N-37 ID5
N-Al ID5
N-Ice ID5

Figure S2

Gel images of DNA

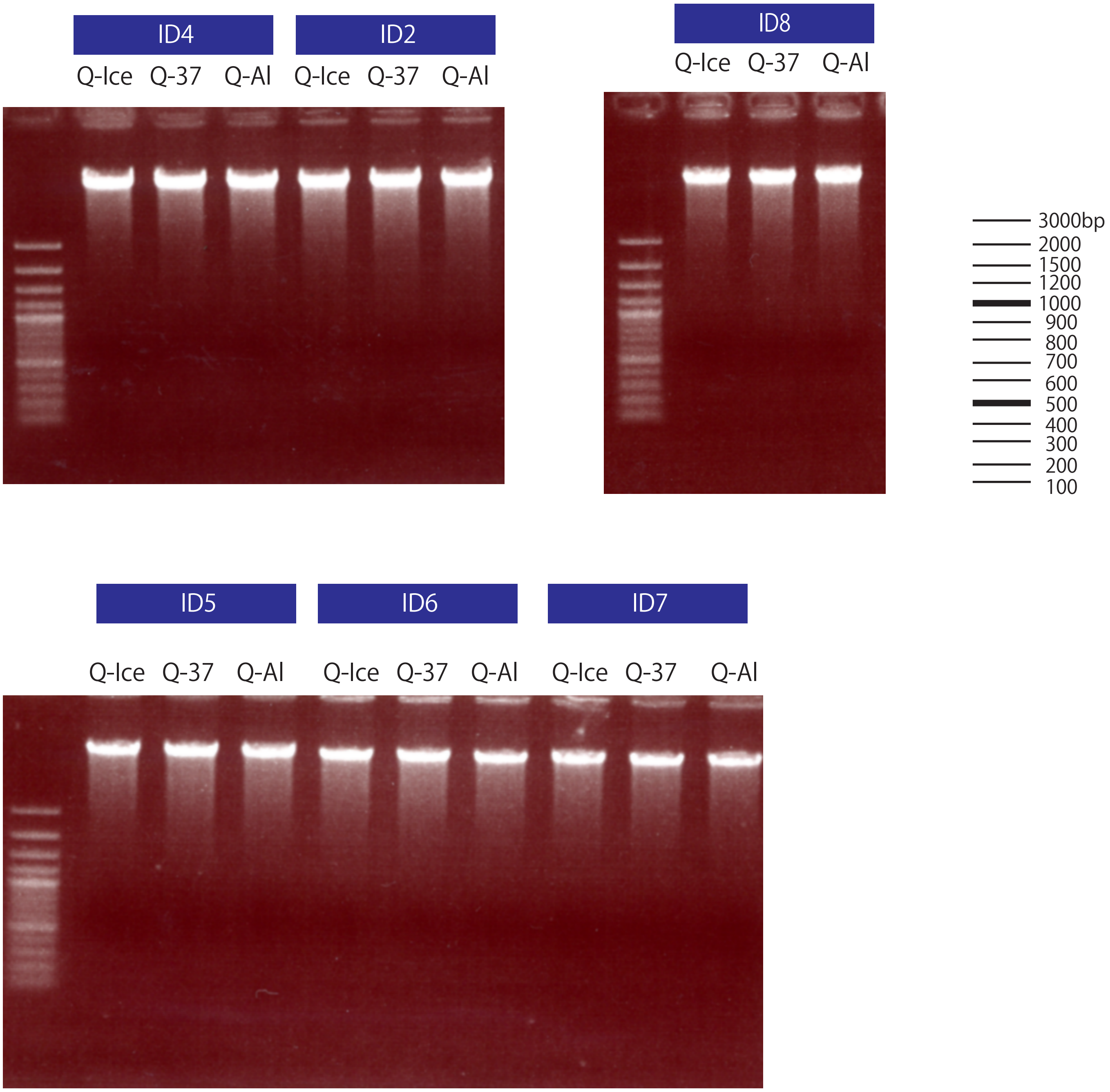

Figure S3 Gel images of PCR products of GAPDH

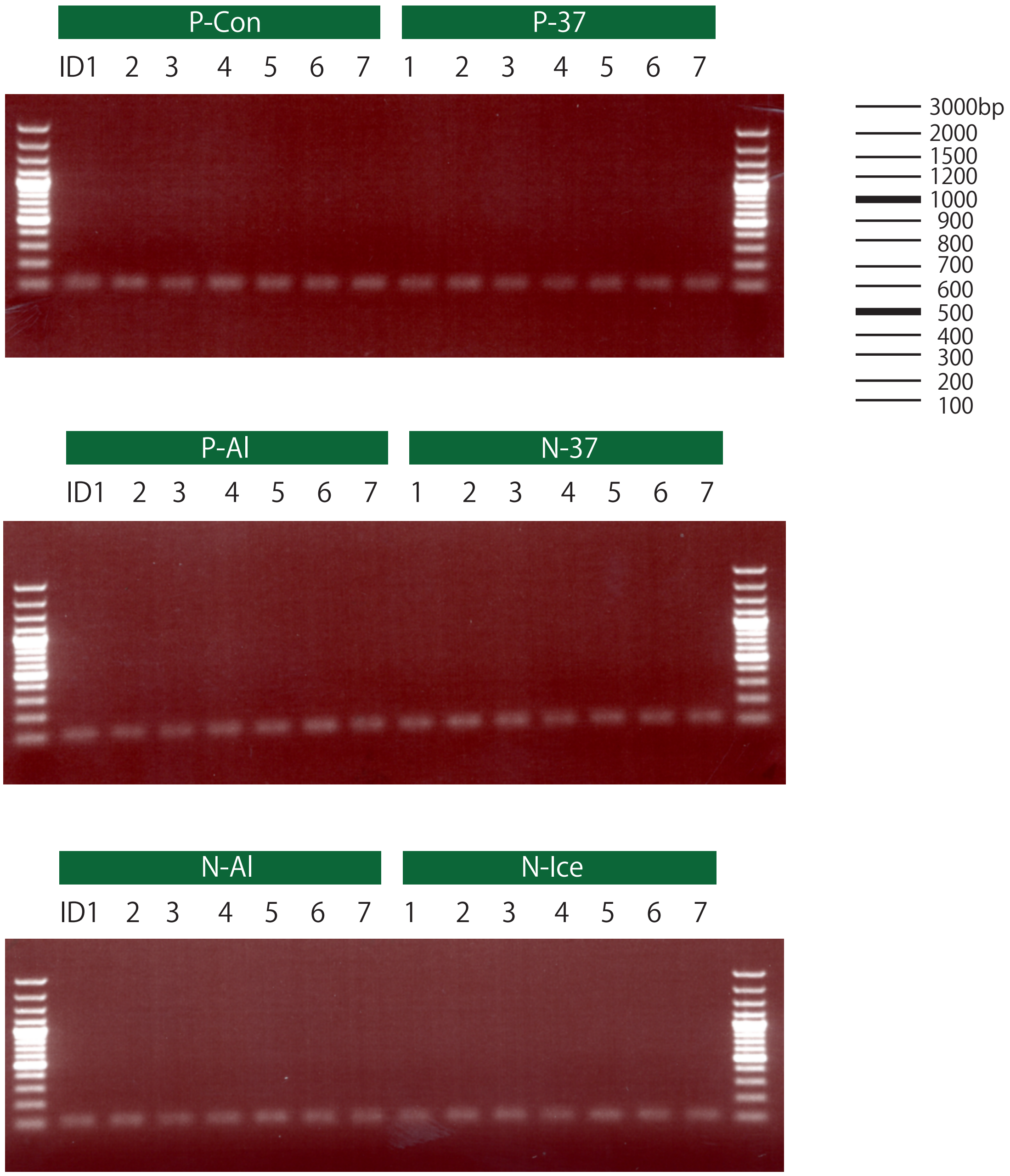

Figure S4 Gel images of PCR products of RPS18

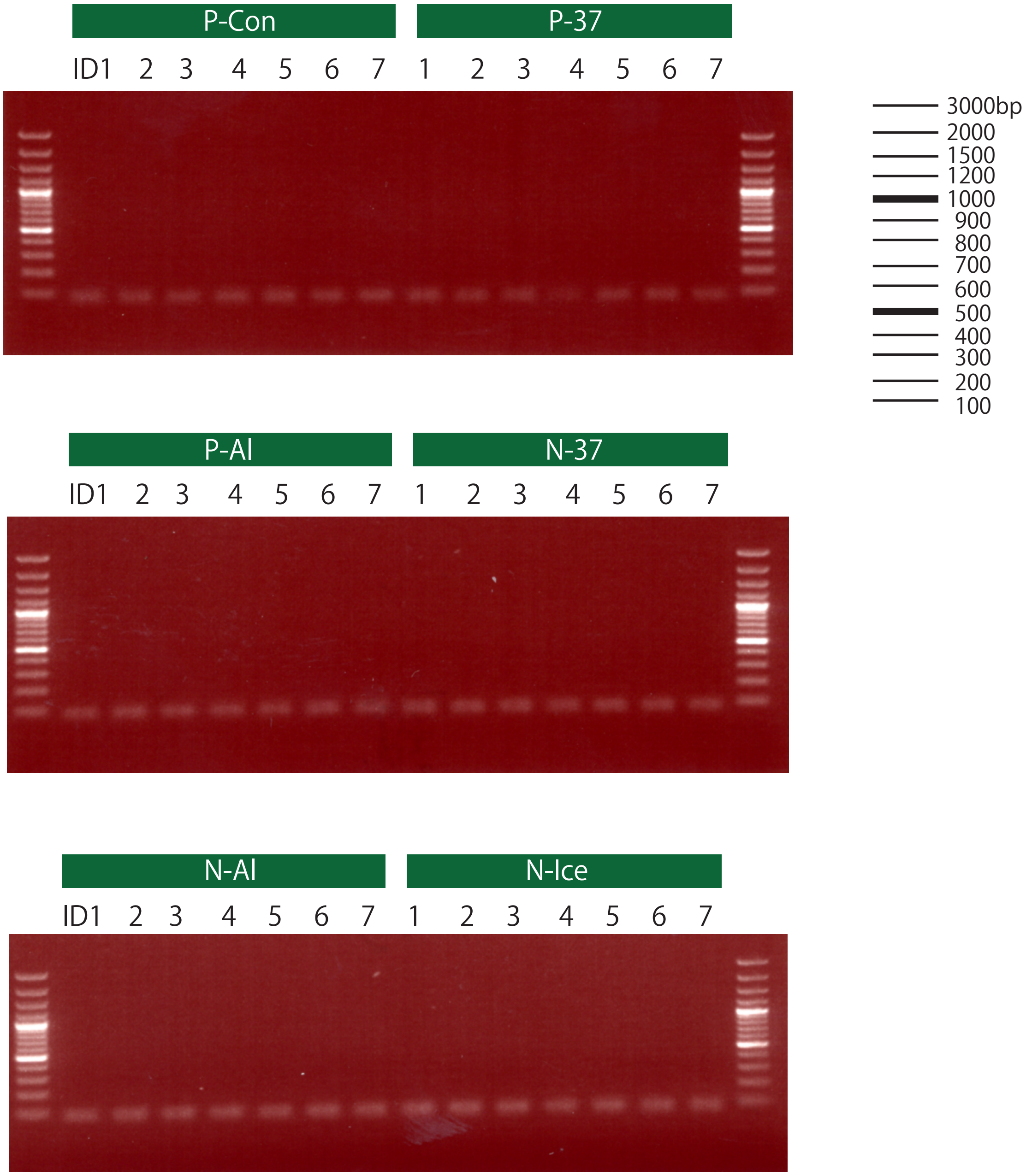

Supplement: Supplementary file 1 — Supplementary Information. [file 41598_2021_96567_MOESM1_ESM.pdf]
